# Applying Latent Profile Analysis to Identify Lifestyle Profiles and Their Association with Loneliness and Quality of Life among Community-Dwelling Middle- and Older-Aged Adults in South Korea

**DOI:** 10.3390/ijerph182312374

**Published:** 2021-11-25

**Authors:** Kang-Hyun Park, Eun-Young Yoo, Jongbae Kim, Ickpyo Hong, Jae-Shin Lee, Ji-Hyuk Park

**Affiliations:** 1 Super-Aged Society New Normal Lifestyle Research Institute, Yonsei University, Wonju 26493, Korea; alcsro44@gmail.com; 2 Department of Occupational Therapy, Yonsei University, Wonju 26493, Korea; splash@yonsei.ac.kr (E.-Y.Y.); jongbae@yonsei.ac.kr (J.K.); ihong@yonsei.ac.kr (I.H.); 3 Department of Occupational Therapy, Konyang University, Daejeon 35365, Korea; jaeshin@konyang.ac.kr

**Keywords:** community-dwelling middle- and older-aged adults, multi-faceted lifestyle, quality of life, mental health, health promotion, latent profile analysis

## Abstract

This study aimed to examine the multi-faceted lifestyle profiles of community-dwelling middle- and older-aged adults based on their physical activity, participation in various activities, and nutrition. It identified the association of lifestyle profiles with demographic variables, quality of life, and mental health. The analysis included 569 participants (mean age = 60.2; SD = 4.3). Latent profile analysis identified three distinctive lifestyle profiles: “inactive and unbalanced” (36.4%), “basic life maintenance” (54.6%), and “active and balanced” (9.1%). Sex (*p* < 0.001), age (*p* < 0.001), and regular medication intake (*p* < 0.01) were statistically significantly different among the three profiles. Of the “inactive and unbalanced” lifestyle group, 63.3% of it was comprised of by females, and a relatively large distribution was aged over 65. In the “basic life maintenance” subgroup, males showed a relatively large distribution, and 92.6% of participants were aged 55–64. People with active and balanced lifestyles demonstrated high quality of life levels (*p* < 0.001) and low loneliness levels (*p* < 0.01). Multinomial logistic regression revealed a statistically significant positive association between lifestyle profiles and quality of life (*p* < 0.001) as well as mental health (*p* < 0.01). Therefore, health promotion that considers multi-faceted lifestyle factors would need to improve health and quality of life among community-dwelling middle- and older-aged adults in South Korea.

## 1. Introduction

Profiling and monitoring multi-faceted lifestyles are essential to establish and develop preventive health interventions and policies [1,2]. A healthy lifestyle, which consists of a high level of physical activity, balanced participation in various activities, and healthy eating habits, has been identified as a major modifiable factor in preventing chronic diseases [3,4], cardiovascular disease [5,6], cognitive decline among the elderly [7,8,9], and mental health issues, such as depression and loneliness [10]. Therefore, a multi-faceted lifestyle is a vital determinant of overall health and quality of life, with evidence suggesting a link between individuals’ lifestyle patterns and the development of various chronic diseases and mental health issues. Hence, it is necessary to evaluate multi-faceted lifestyles and categorize lifestyles by analyzing individuals’ lifestyle patterns. Recognizing the types of individuals’ lifestyles makes it possible to implement preventive interventions for health and well-being according to their lifestyles. However, previous studies have tended to conduct lifestyle interventions without considering personal lifestyle patterns. In order to develop lifestyle interventions, the identification of lifestyle patterns should be performed. Therefore, identifying the lifestyle patterns of community-dwelling elderly is a public health imperative to support lifestyle interventions, which can improve healthy and successful aging.

Population aging is occurring worldwide. In South Korea, 14.9% of the population was aged 65 years or above in 2019; by 2026, it is expected to become a super-aged society when the proportion of the population that is elderly reaches 20% [11]. Aging represents a major challenge because it can influence the development of countries and bring about economic and social change [12]. In this super-aging society, it is a fact that improving the health and quality of life of the elderly is vital to relieve the intense pressure brought by an aging society [13,14].

Research is beginning to focus on lifestyle patterns of the elderly, and a few observational studies have examined the lifestyles of the elderly population [15,16,17]. However, these studies have assessed only specific lifestyle behaviors and did not undertake a multidimensional lifestyle factor approach. Whereas most previous studies have focused on exercise, nutrition, and smoking or drinking habits, a recent study showed that participation in meaningful activities, such as daily living, work, leisure, and social participation, are important lifestyle factors that improve the elderly population’s quality of life [18,19].

Moreover, data-driven statistical methods, such as k-means cluster analysis, are commonly used to identify lifestyle patterns [20,21]. However, this method has limitations, because the choice of cluster criteria for k-means cluster analysis is arbitrary [22]. Latent profile analysis (LPA) addresses this limitation. It is a probability-based approach that classifies participants into discrete clusters based on their distinctive response patterns and allows for the statistical comparison of models to determine the number of clusters [22,23].

To bridge this research gap, we empirically examined the multi-faceted lifestyle profiles of community-dwelling middle- and older-aged adults in South Korea using LPA. We utilized a lifestyle questionnaire to gather data on multi-faceted lifestyle factors, including physical activity, nutrition, and participation in meaningful activities. Finally, we examined the association between lifestyle and sociodemographic variables, quality of life, and mental health.

## 2. Methods

### 2.1. Participants

This study was designed as a cross-sectional online survey, conducted by a specialized online research company (www.embrain.com). The company has 1,428,252 research panels, which are made up of respondents who have previously announced their intention to participate in surveys and provided personal information through a contract with the research company. This study targeted elderly participants; the inclusion criteria were as follows: (1) community-dwelling middle- and older-aged adults aged over 55 years and (2) having lived in South Korea in recent years. Individuals who agreed to participate checked the consent tick box on the first page of the survey form. This study was conducted between August and November 2020, with a total of 569 respondents. It was approved by the Institutional Review Board of Yonsei University Mirae Campus (1041849-202103-SB-039-03).

### 2.2. Measures

#### 2.2.1. Lifestyle Factors

Lifestyle was measured using the Yonsei lifestyle profile (YLP) questionnaire [19,24]. The YLP questionnaire is comprised of 60 items that measure three different lifestyle factors: (1) physical activity, (2) activity participation, and (3) nutrition. The YLP demonstrated high internal reliability, with a Cronbach’s alpha of 0.83. The intraclass correlation coefficient was 0.97 for the total score of the YLP regarding test–retest reliability [24]. In this study, three items of physical activity, five items of activity participation, and three items of nutrition were used from the YLP questionnaire to derive a potential class that comprehensively includes the multi-faceted lifestyle of middle- and older-aged adults. The questionnaire is provided in Supplementary File S1 which included the YLP questionnaire.

Physical activity. A 5-point Likert scale was used to assess the frequency of respondents’ participation in three different physical activities. For example, participants were asked “How often did you participate in low-intensity physical activity in the last week?” Participants answered these questions using a Likert scale: (1) never, (2) 1–2 times per week, (3) 3–4 times per week, (4) 5–6 times per week, and (5) every day. According to the American College of Sports Medicine (ACSM), physical activity can be divided into three types based on intensity [25]. The three physical activities included in the questionnaire were: low-intensity physical activity equivalent to 2–2.9 metabolic equivalent of task (MET), such as gardening and house cleaning; moderate-intensity physical activity equivalent to 3–5.9 MET, including swimming and doubles tennis; and high-intensity physical activity equivalent to 6–9.9 MET, such as running and climbing, in addition to walking exercises.

Activity participation. Five items were used to measure the frequency of participation in various pursuits, such as activities of daily living (ADL), leisure, social activity, work, and education, using a 5-point Likert scale. A higher score indicated more frequent participation in various activities over a week. 

Nutrition. To measure the daily diet pattern of community-dwelling middle- and older-aged adults, a total of three items were used. Participants were required to answer questions regarding their carbohydrate, protein, and fat intake over a week. The items of nutrition were also measured on a 5-point Likert scale; the higher the score, the more the nutrients were consumed.

#### 2.2.2. Quality of Life

The World Health Organization Quality of Life-Brief (WHOQOL-BREF) was used to measure the quality of life level [26,27]. The measurement consists of 26 items, and there are four major domains: physical, psychological, social, and environmental. The items were rated on a 5-point Likert scale and the raw domain scores were converted to a scale ranging from 0 to 100 to facilitate comparison with other instruments, with higher scores indicating a higher quality of life [28]. Cronbach’s alpha was 0.92 in this study.

#### 2.2.3. Loneliness

To evaluate participants’ mental health, loneliness was measured using the Korean Geriatric Loneliness Scale (KGLS) [29]. This tool multidimensionally measures the loneliness of the elderly across family, friends, and social domains. The KGLS consists of 14 items rated on a 4-point Likert scale. The score of the tool ranges from 14 to 56, with higher scores indicating higher levels of loneliness [29]. Cronbach’s alpha was 0.61 in this study.

### 2.3. Data Analysis

Descriptive analyses were conducted to understand the means and standard deviations of all variables using SPSS version 22 (IBM, Armonk, NY, USA). To identify and describe the lifestyle profiles of community-dwelling middle- and older-aged adults, LPA was used for analysis using Mplus 8.4 (Muthén & Muthén, Los Angeles, CA, USA) [15]. To conduct this analysis and obtain latent profiles, the following lifestyle factors were used: low-intensity, moderate-intensity, and high-intensity physical activity, ADL, leisure, social activity, work, and education, as well as carbohydrate, protein, and fat intake. Missing data were imputed for the LPA using full-information maximum likelihood estimation [30,31].

Initially, we started with a two-profile solution and increased the number of extracted profiles until the model fit no longer improved [32]. To determine the optimal number of latent profiles, statistical and theoretical criteria were comprehensively considered [32]. Model fit was assessed using Akaike information criterion (AIC) statistics, the Bayesian information criterion (BIC), and the sample-size-adjusted Bayesian information criterion (SS-ABIC), with lower values indicating a better fit. The Lo–Mendell–Rubin-adjusted likelihood ratio test (LMR-LRT) was also used as a statistical criterion to compare k group models to k^−1^ group models, where the simpler model is the null model, and show that lower values have a better model fit [33]. Additionally, entropy, which is defined as the accuracy of individual assignments to different groups, was assessed. For the entropy, values higher than 0.70 and close to 1 are preferred [34].

Univariate analysis of categorical variables was performed using the chi-square test. Multinomial logistic regression was performed to test the association between the identified lifestyle latent profiles (reference group = class 1) and any other variables. A *p*-value < 0.05 was regarded as statistically significant.

## 3. Results

### 3.1. Characteristics of the Study Population

The sociodemographic characteristics of the participants are presented in Table 1. Of the study participants, there was an even sex distribution, with 50.4% males and 49.6% females. Regarding age groups, individuals aged 55–64, 65–74, and over 75 made up 84.7%, 14.9%, and 0.4% of the sample, respectively. The mean age was 60.2 years with a standard deviation (SD) of 4.3. The majority of the participants were educated beyond college (69.2%) and lived in metropolitan areas (53.4%) (Table 1).

### 3.2. Lifestyle Profile Models by Latent Profile Analysis

To identify profiles according to the 11 lifestyle factors, a latent profile analysis was conducted (Table 2). As the number of latent profiles increased from two to four, the AIC, BIC, and SS-ABIC gradually decreased from one- to four-class models. Additionally, higher entropy was demonstrated in all models. However, the *p*-value of the LMR-LRT in the four-class model was 0.578, meaning that the four-class model was rejected and a three-class model (simpler model) was adopted. Therefore, by comprehensively considering the various statistical criteria above, a model with three latent profiles was selected as the optimal model.

**Figure 1:**
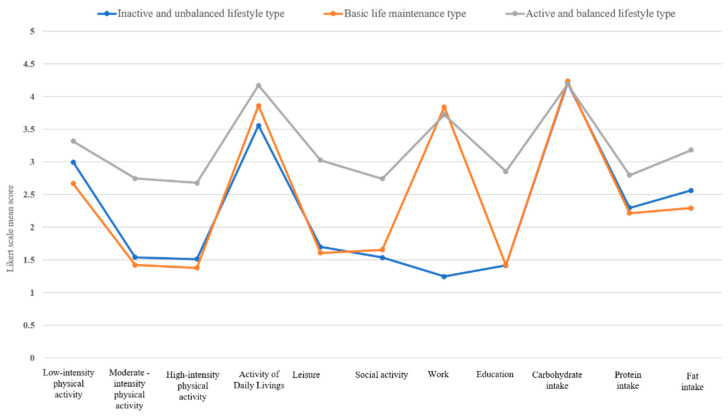
Three lifestyle profiles by 11 lifestyle factors.

**Figure 2:**
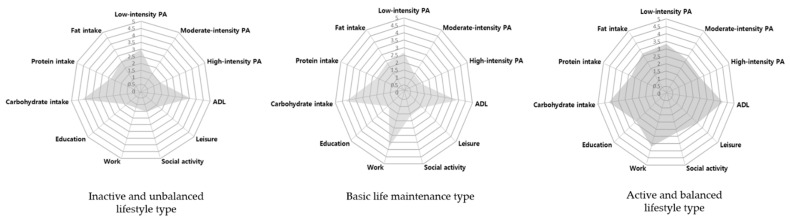
The tendencies of three distinctive lifestyle types. PA: Physical activity; ADL: Activity of Daily Livings.

Regarding lifestyle patterns, community-dwelling middle- and older-aged adults were classified into three profiles: “inactive and unbalanced lifestyle type”, “basic life maintenance type”, and “active and balanced lifestyle type” (Figure 1). In Figure 1, the y-axis is the Likert scales of each lifestyle factor and the x-axis is the lifestyle factors. Figure 2 illustrates the tendencies of the three distinctive lifestyle types.

The “inactive and unbalanced lifestyle type” profile consisted of 36.4% (N = 207) of the participants. They demonstrated low levels of physical activity and participation in other meaningful activities compared to participants in the other profiles. Regarding nutrition, their protein and fat intake scores were also lower than others’.

The “basic life maintenance type” profile was the largest subgroup, which included 54.5% (N = 309) of the total participants. Participants with this profile demonstrated high scores in basic life maintenance activities such as ADL, work, and carbohydrate intake compared to other lifestyle factors.

The “active and balanced lifestyle type” profile was the smallest group, comprising only 9.1% (N = 52) of the participants. In contrast to other profiles, the participants in this group showed overall high scores for most lifestyle factors, including physical activities, activity participation, and nutrition, compared to participants in the other profiles. They tended to participate in regular physical activities and various meaningful activities in daily life as well as consume a balanced diet.

### 3.3. Characteristics of the Identified Lifestyle Profiles

The demographic characteristics of the three lifestyle profiles are shown in Table 3. There were statistically significant differences in gender, age, and regular medication intake among the three profiles. In the “inactive and unbalanced lifestyle type” subgroup, 63.3% were females and those aged over 65 years of age showed a relatively large distribution. Compared with other lifestyle profiles, males showed a relatively large distribution in the “basic life maintenance type” subgroup, and participants aged 55–64 years accounted for 92.6% of this subgroup. For the “active and balanced lifestyle type”, the ratio of male to female was evenly distributed.

Table 4 presents the results of the profile comparisons of loneliness (KGLS) and quality of life (WHOQOL). Statistically significant differences were observed among the three profiles. The level of loneliness was lower in profile 3, which was the “active and balanced lifestyle type”, in comparison to profiles 1 and 2. The average quality of life was higher in profile 3 than in the other profiles.

### 3.4. Association between Lifestyle Profiles and Quality of Life and Mental Health

To analyze the association between lifestyle profiles and other variables, multinomial logistic regression analyses were conducted (Table 5). A *p*-value < 0.05 was regarded as statistically significant. Analyses were conducted with each of the three latent profiles as the reference group to determine how each variable contributed to the composition and differentiation between the latent profiles. Middle- and older-aged adults of higher age (*p* < 0.05) and lower quality of life (*p* < 0.001) were more likely to fall in the “inactive and unbalanced lifestyle type” rather than the “active and balanced lifestyle type”. Furthermore, people with a lower quality of life level (*p* < 0.001) were more likely to be the “basic life maintenance type” compared to the “active and balanced lifestyle type”. Being male (*p* < 0.001) and of higher age (*p* < 0.001) were related to classification as a member of the “inactive and unbalanced lifestyle type” rather than the “basic life maintenance type”.

## 4. Discussion

This study aimed to use multi-faceted lifestyle factors to identify lifestyle profiles of community-dwelling middle- and older-aged adults using LPA and examined demographic characteristics, mental health, and quality of life associated with lifestyle profiles. It illustrated that community-dwelling middle- and older-aged adults in South Korea can be classified into three distinct lifestyle profiles: “inactive and unbalanced lifestyle type” (36.4%), “basic life maintenance type” (54.5%), and “active and balanced lifestyle type” (9.1%). The findings of this study demonstrate that older adults in the community are not a homogeneous group; rather, there are substantial dissimilarities in terms of lifestyle patterns.

In 2011, Heroux et al. [15] found that two lifestyle classes exist by using four indicators (diet, fitness, smoking, and drinking) through latent class analysis. Results from this study showed that participants in class 1 were characterized by a higher probability of partaking in each of the four unhealthy lifestyle behaviors than participants in class 2. In 2014, Södergren et al. [30] supported these findings; they examined the lifestyle patterns of retirement-age adults and found two distinctive classes: healthy lifestyle and less healthy lifestyle. Eating habits, physical activity, sedentary behaviors, and tobacco and alcohol consumption were the indicators in that study [30]. Previous studies have analyzed lifestyle patterns by using limited factors such as exercise, diet, smoking, and drinking [15,30]. However, as lifestyle is characterized by various factors such as physical activity, activity participation, and nutrition [18,19,31], other lifestyle indicators should be included to understand multi-faceted lifestyle patterns. Thus, in this study, 11 factors were included to identify the lifestyle profiles of middle- and older-aged adults, and three multi-faceted lifestyle profiles were identified.

Profile 1, the “inactive and unbalanced lifestyle type”, comprised mainly of females aged 55 to 64 years, and was characterized by physical inactivity, low levels of activity participation, and lower nutrient intake. In contrast, profile 2, the “basic life maintenance type”, comprised mostly of males aged between 55 and 64 years who have a high level of education. From these results, it can be shown that sex and age are important factors that can affect personal lifestyle patterns. Moreover, these findings were revealed from previous studies that demonstrated the association between sex, age, and lifestyle patterns [35,36,37].

Regarding the association between lifestyle profiles and quality of life and mental health, our findings are consistent with previous studies that have examined lifestyles of the elderly [18,33,34]. In terms of physical activity as one of the lifestyles, much of the literature has illustrated that participating in physical activity regularly and actively is able to increase significantly quality of life and mental health in older adults [38,39,40]. In addition, in the present study as well, profile 3, which participated in various physical activities actively, demonstrated a statistically significant higher score for quality of life compared to other groups. These findings suggest that encouraging middle- and older-aged adults to participate in various physical activities in their daily life is essential to improve their quality of life [41,42].

This study found that community-dwelling middle- and older-aged adults in profile 3 have a higher level of quality of life compared to those in the other groups. These findings reinforce studies in the literature that suggest a healthy lifestyle is positively related to quality of life [18,33,34]. Regarding loneliness, people in profile 3 demonstrated a lower level of loneliness than those in the other profiles. Previous studies also found that an unhealthy lifestyle is negatively related to loneliness [43,44]. Therefore, this study showed that Quality of Life (QoL) and loneliness are associated with the lifestyle profiles of community-dwelling middle- and older-aged adults.

This study makes it possible to establish several suggestions in terms of public health. It has demonstrated the importance of considering factor groupings in preventive health interventions. When considering the association between quality of life, loneliness, and multi-faceted lifestyles, focusing on factors such as exercise, diet, smoking, and alcohol consumption no longer seems to be sufficient when developing public health services for community-dwelling middle- and older-aged adults. Therefore, it is necessary to consider multi-faceted lifestyles to improve mental health and quality of life. Similarly, differences in demographic characteristics should also be considered. For example, this study shows that middle- and older-aged females are more vulnerable to inactive and unbalanced lifestyles. It also provides information on the classification of community-dwelling middle- and older-aged adults, which could be used to classify and study the middle- and older-aged adult population. By using LPA, organizations, health policymakers, and other researchers can understand different lifestyle profiles, and develop and establish target interventions as well as distribute appropriate resources.

However, the findings of this study do have limitations and should be interpreted carefully. First, it is an exploratory cross-sectional study that does not reflect trends over time; thus, further studies over a longer period should be conducted. Second, the number of people in this study was relatively small, indicating the need for caution in generalizing the results. Lastly, this study relied on self-reported measures, which may be susceptible to various errors and biases, such as memory bias. Moreover, due to the lack of questions on sociodemographic information in the questionnaire, its capacity to analyze that what factors influenced the division of these groups was limited. Thus, future studies should utilize other additional data sources, such as objective measurements conducted by professionals, and include more detailed sociodemographic questions to improve the validity and objectivity of the results.

## 5. Conclusions

This study found that there are specific profiles of community-dwelling middle- and older-aged adults according to their daily lifestyles. The vast majority of the middle- and older-aged adults demonstrated low levels of physical activity and meaningful activity participation, in addition to low consumption of necessary nutrients. Additionally, this showed a lower level of quality of life and loneliness compared to people in the active and balanced groups. These findings can be used to identify lifestyle profiles and guide the development of specific interventions to improve the quality of life and loneliness of community-dwelling middle- and older-aged adults.

## Figures and Tables

**Table 1:** General characteristics (N = 569).

Variables		N	(%)
Sex	Male	287	50.4
	Female	282	49.6
Age (Mean ± SD: 60.2 ± 4.3)	55–64	482	84.7
	65–74	85	14.9
	>75	2	0.4
Educational attainment	Elementary school	4	0.7
	Middle school	9	1.6
	High school	161	28.3
	College or university	395	69.4
Residential area	Metropolitan	304	53.4
	Provincial big city	116	20.4
	Small and medium-sized cities	139	24.4
	Countryside	10	1.8
Regular medication intake	Yes	291	51.1
	No	278	48.9

**Table 2:** Model fit statistics of latent profile models (N = 569).

No. of Profiles	AIC	BIC	SS-ABIC	LMR-LRT	Entropy	Latent Profile Proportions (%)			
						1	2	3	4
2	16,869.422	17,017.114	16,909.179	503.666 ***	0.904	87.170	12.830		
3	16,552.489	16,758.307	16,606.278	336.512 ***	0.956	36.380	54.482	9.139	
4	16,404.205	16,656.150	16,472.026	170.050	0.940	30.053	8.436	54.657	6.854

Abbreviations: AIC, Akaike information criterion; BIC, Bayesian information criterion; SS-ABIC, sample-sized-adjusted Bayesian information criterion; and LMR-LRT, Lo–Mendell–Rubin-adjusted likelihood ratio test. *** *p* < 0.001.

**Table 3:** Demographic characteristics of identified three different lifestyle profiles.

Variables		Latent Profile (N (%))			*χ* ^2^
		Profile 1	Profile 2	Profile 3	
Sex	Male	76 (36.7)	190 (61.3)	21 (40.4)	32.302 ***
	Female	131 (63.3)	120 (38.7)	31 (59.6)	
Age	55–64	150 (72.5)	287 (92.6)	45 (86.5)	40.006 ***
	65–74	55 (26.5)	23 (7.4)	7 (13.5)	
	>75	2 (1.0)	-	-	
Educational attainment	Elementary school	2 (1.0)	2 (0.6)	-	6.593
	Middle school	3 (1.4)	5 (1.6)	1 (1.9)	
	High school	67 (32.4)	77 (24.8)	17 (32.7)	
	College or university	134 (64.7)	226 (72.9)	34 (65.4)	
	No response	1 (0.5)	-	-	
Residential area	Metropolitan	103 (49.8)	177 (57.1)	24 (46.2)	11.501
	Provincial big city	53 (25.6)	52 (16.8)	11 (21.2)	
	Medium-sized city	45 (21.7)	77 (24.8)	17 (32.7)	
	Countryside	6 (2.9)	4 (1.3)	-	
Regular medication intake	Yes	122 (58.9)	141 (45.5)	28 (53.8)	9.158 **
	No	85 (41.1)	169 (54.5)	24 (46.2)	

Profile 1: inactive and unbalanced lifestyle type; profile 2: basic life maintenance type; and profile 3: active and balanced lifestyle type. ** *p* < 0.01; and *** *p* < 0.001.

**Table 4:** Profiles comparison on mental health and quality of life.

Variables	Latent Profile (M (SD))			*F*
	Profile 1	Profile 2	Profile 3	
Loneliness (KGLS)	35.63 (3.83)	35.53 (3.68)	37.54 (4.94)	6.14 **
Quality of life (WHOQOL)	82.17 (16.05)	83.00 (15.78)	92.50 (16.20)	9.17 ***

Profile 1: inactive and unbalanced lifestyle type; profile 2: basic life maintenance type; and profile 3: active and balanced lifestyle type. ** *p* < 0.01; and *** *p* < 0.001.

**Table 5:** Association between lifestyle profiles and other variables.

Variables		B	SE	OR	96% CI
Profile 3 vs.					
Profile 1	Sex	−0.155	0.317	0.856	0.460–1.595
	Age	0.092 *	0.038	1.096	1.017–1.180
	Educational attainment	−0.035	0.271	0.966	0.568–1.644
	Residential area	−0.106	0.170	0.899	0.645–1.254
	Regular medication intake	0.207	0.312	1.230	0.667–2.268
	Loneliness (KGLS)	−0.073 **	0.026	0.929	0.883–0.978
	Quality of life (WHOQOL)	−0.043 ***	0.011	0.957	0.938–0.978
Profile 2	Sex	0.849 **	0.306	2.337	1.284–4.256
	Age	−0.074	0.038	0.929	0.862–1.002
	Educational level	0.221	0.267	1.247	0.739–2.105
	Residential area	−0.202	0.165	0.817	0.592–1.128
	Regular medication intake	−0.335	0.301	0.715	0.397–1.289
	Loneliness (KGLS)	−0.064 *	0.025	0.938	0.892–0.985
	Quality of life (WHOQOL)	−0.040 ***	0.010	0.961	0.941–0.980
Profile 2 vs.					
Profile 1	Sex	−1.004 ***	0.185	0.366	0.255–0.527
	Age	0.165 ***	0.024	1.180	1.126–1.236
	Educational level	−0.255	0.163	0.775	0.562–1.067
	Residential area	0.096	0.101	1.100	0.902–1.342
	Regular medication intake	0.543 **	0.182	1.720	1.205–2.456
	Loneliness (KGLS)	−0.009	0.014	0.991	0.964–1.019
	Quality of life (WHOQOL)	−0.003	0.006	0.997	0.986–1.008

*Note*. Reference group: gender (female). Regular medication intake (no). Profile 1: inactive and unbalanced lifestyle type; profile 2: basic life maintenance type; and profile 3: active and balanced lifestyle type. B = the unstandardized beta, SE = Standard error, OR = odds ratio, CI = confidence interval. * *p* < 0.05; ** *p* < 0.01; and *** *p* < 0.001.

## Supplementary Materials

The following are available online at https://www.mdpi.com/article/10.3390/ijerph182312374/s1, File S1: Yonsei Lifestyle Profile (YLP) questionnaire.

## References

[1] Ministério da Saúde Brasília-DF (2011). Plano de Ações Estratégicas Para o Enfrentamento das Doenças Crônicas não Transmissíveis (Dcnt) No Brasil 2011–2022.

[2] World Health Organization (2013). Global Action Plan for the Prevention and Control of NCDs 2013–2020.

[3] Bauman A.E. (2004). Updating the Evidence that Physical Activity is Good for Health: An Epidemiological Review 2000–2003. J. Sci. Med. Sport..

[4] Dunstan D.W., Thorp A.A., Healy G.N. (2011). Prolonged Sitting: Is It a Distinct Coronary Heart Disease Risk Factor?. Curr. Opin. Cardiol..

[5] Kurth T., Moore S., Gaziano M., Kase C., Stampfer M.J., Berger K., Buring J.E. (2006). Healthy lifestyle and the risk of stroke in women. Arch. Intern. Med..

[6] Van Dam R.M., Li T., Spiegelman D., Franco O.H., Hu F.B. (2008). Combined Impact of Lifestyle Factors on Mortality: Prospective Cohort Study in US Women. BMJ.

[7] Anstey K.J., Bahar-Fuchs A., Herath P., Kim S., Burns R., Rebok G.W., Cherbuin N. (2015). Body Brain Life: A Randomized Controlled Trial of an Online Dementia Risk Reduction Intervention in Middle-aged Adults at Risk of Alzheimer’s Disease. Alzheimer’s Dement..

[8] McMaster M., Kim S., Clare L., Torres S.J., Cherbuin N., D’Este C., Anstey K.J. (2020). Lifestyle Risk Factors and Cognitive Outcomes from the Multidomain Dementia Risk Reduction Randomized Controlled Trial, Body Brain Life for Cognitive Decline (BBL-CD). J. Am. Geriatr. Soc..

[9] Ngandu T., Lehtisalo J., Solomon A., Levälahti E., Ahtiluoto S., Antikainen R., Bäckman L., Hänninen T., Jula A., Laatikainen T. (2015). A 2 Year Multidomain Intervention of Diet, Exercise, Cognitive Training, and Vascular Risk Monitoring versus Control to Prevent Cognitive Decline in At-Risk Elderly People (FINGER): A Randomised Controlled Trial. Lancet.

[10] Nho J., Yoo S. (2018). Relationships among Lifestyle, Depression, Anxiety, and Reproductive Health in Female University Students. Korean J. Women Health Nurs..

[11] Statistics Korea (2019). Population Projections for Korea: 2017–2067.

[12] Pan W.C., Ma Q., Sun H.P., Xu Y., Luo N., Wang P. (2017). Tea Consumption and Health-Related Quality of Life in Older Adults. J. Nutr. Health Aging.

[13] World Health Organization (2002). Active Ageing: A Policy Framework.

[14] The State Council Information Office, China (2006). The Development of China’s Undertakings for the Aged. http://www.china.org.cn/english/China/191990.htm.

[15] Heroux M., Janssen I., Lee D.C., Sui X., Hebert J.R., Blair S.N. (2011). Clustering of Unhealthy Behaviors in the Aerobics Center Longitudinal Study. Prev. Sci..

[16] Laaksonen M., Prattala R., Karisto A. (2001). Patterns of Unhealthy Behaviour in Finland. Eur. J. Public Health.

[17] Shi L., Morrison J.A., Wiecha J., Horton M., Hayman L.L. (2011). Healthy Lifestyle Factors Associated with Reduced Cardio Metabolic Risk. Br. J. Nutr..

[18] Park K., Park J. (2019). Analysis of Convergent Influence of Functional Level, Environmental Factors and Lifestyle on Health and Quality of Life among Elderly Using Structural Equation Model. J. Korea Converg. Soc..

[19] Park K.H., Park J.H. (2020). Development of an Elderly Lifestyle Profile: A Delphi Survey of Multidisciplinary Health-Care Experts. PLoS ONE.

[20] Lee E., Shin J. (2004). A Study on Lifestyle-Based Market Segmentation of the Korean Mature Consumers. J. Korean Gerontol. Nurs..

[21] Park Y.S., Kim H. (2016). Gender Differences in Healthy Lifestyle Cluster and Their Relationship with Depressive Symptoms among Middle-Aged and Older Adults in Korea. Korean J. Health Edu. Promot..

[22] Todd M., Adams M.A., Kurka J., Conway T.L., Cain K.L., Buman M.P., Frank L.D., Sallis J.F., King A.C. (2016). Gis-Measured Walkability, Transit, and Recreation Environments in Relation to Older Adults’ Physical Activity: A Latent Profile Analysis. Prev. Med..

[23] Berlin K.S., Williams N.A., Parra G.R. (2014). An Introduction to Latent Variable Mixture Modeling (Part 1): Overview and Cross-Sectional Latent Class and Latent Profile Analyses. J. Pediatr. Psychol..

[24] Park K., Han D., Park J. The Yonsei Lifestyle Profile (YLP) for adults and the older adults: Development and test-retest reliability. Proceedings of the 2020 The Korean Gerontological Society Annual Conference.

[25] American College of Sports Medicine (2014). ACSM’s Guidelines for Exercise Testing and Prescription.

[26] Group WHOQOL (1998). Development of the World Health Organization WHOQOL-BREF quality of life assessment. Psychol. Med..

[27] Skevington S.M., Lotfy M., O’Connell K.A. (2004). The World Health Organization’s WHOQOL-BREF Quality of Life Assessment: Psychometric Properties and Results of the International Field Trial. A Report from the WHOQOL Group. Qual. Life Res..

[28] Min S.K., Kim K.I., Lee C.I., Jung Y.C., Suh S.Y., Kim D.K. (2002). Development of the Korean Versions of WHO Quality of Life Scale and WHOQOL-Bref. Qual. Life Res..

[29] Lee S.E. (2019). Development of the Korean Geriatric Loneliness Scale (KGLS). J. Korean Acad. Nurs..

[30] Södergren M., Wang W.C., Salmon J., Ball K., Crawford D., McNaughton S.A. (2014). Predicting Healthy Lifestyle Patterns among Retirement Age Older Adults in the Well Study: A Latent Class Analysis of Sex Differences. Maturitas.

[31] Vajdi M., Nikniaz L., Pour Asl A.M., Abbasalizad Farhangi M. (2020). Lifestyle Patterns and Their Nutritional, Socio-Demographic and Psychological Determinants in a Community-Based Study: A Mixed Approach of Latent Class and Factor Analyses. PLoS ONE.

[32] Park K., Won K., Park J. (2019). A Systematic Study on the Multifaceted Lifestyle Assessment Tools for Community-Dwelling Elderly: Trend and Application Prospect. Thera. Sci. Neurorehab..

[33] Ferreira L.K., Meireles J.F.F., Ferreira M.E.C. (2018). Evaluation of Lifestyle and Quality of Life in the Elderly: A Literature Review. Rev. Bras. Geriatr. Gerontol..

[34] Footit J., Anderson D. (2012). Associations between Perception of Wellness and Health-Related Quality of Life, Comorbidities, Modifiable Lifestyle Factors and Demographics in Older Australians. Australas. J. Ageing.

[35] Denton M., Walters V. (1999). Gender Differences in Structural and Behavioral Determinants of Health: An Analysis of the Social Production of Health. Soc. Sci. Med..

[36] Uitenbroek D.G., Kerekovska A., Festchieva N. (1996). Health Lifestyle Behaviour and Socio-Demographic Characteristics. A Study of Varna, Glasgow and Edinburgh. Soc. Sci. Med..

[37] Vari R., Scazzocchio B., D’Amore A., Giovannini C., Gessani S., Masella R. (2016). Gender-Related Differences in Lifestyle May Affect Health Status. Ann. Ist. Super. Sanita.

[38] Syed-Abdul M.M., McClellan C.L., Parks E.J., Ball S.D. (2021). Effects of a Resistance Training Community Programme in Older Adults. Ageing Soc..

[39] Reid K.J., Baron K.G., Lu B., Naylor E., Wolfe L., Zee P.C. (2010). Aerobic Exercise Improves Self-Reported Sleep and Quality of Life in Older Adults with Insomnia. Sleep Med..

[40] Baker B.S., Syed-Abdul M.M., Weitzel K.J., Ball S.D. (2021). Acute Resistance Training May Have Lasting Benefit to Middle-Aged Adults. Gerontol. Geriatr. Med..

[41] Syed-Abdul M.M. (2021). Benefits of Resistance Training in Older Adults. Curr. Aging Sci..

[42] Choi Y.J., Kim C., Park Y.S. (2007). The Effect of Nutrition Education Program in Physical Health, Nutritional Status and Health-Related Quality of Life of the Elderly in Seoul. J. Nutr. Health.

[43] Hawkley L.C., Burleson M.H., Berntson G.G., Cacioppo J.T. (2003). Loneliness in Everyday Life: Cardiovascular Activity, Psychosocial Context, and Health Behaviors. J. Pers. Soc. Psychol..

[44] Richard A., Rohrmann S., Vandeleur C.L., Schmid M., Barth J., Eichholzer M. (2017). Loneliness Is Adversely Associated with Physical and Mental Health and Lifestyle Factors: Results from a Swiss National Survey. PLoS ONE.

